# Sequence Analysis and Molecular Characterization of *Clonorchis sinensis* Hexokinase, an Unusual Trimeric 50-kDa Glucose-6-Phosphate-Sensitive Allosteric Enzyme

**DOI:** 10.1371/journal.pone.0107940

**Published:** 2014-09-18

**Authors:** Tingjin Chen, Dan Ning, Hengchang Sun, Ran Li, Mei Shang, Xuerong Li, Xiaoyun Wang, Wenjun Chen, Chi Liang, Wenfang Li, Qiang Mao, Ye Li, Chuanhuan Deng, Lexun Wang, Zhongdao Wu, Yan Huang, Jin Xu, Xinbing Yu

**Affiliations:** 1 Department of Parasitology, Zhongshan School of Medicine, Sun Yat-sen University, Guangzhou, Guangdong, China; 2 Key Laboratory for Tropical Diseases Control, Sun Yat-sen University, Ministry of Education, Guangzhou, Guangdong, China; 3 The Third Affiliated hospital, Sun Yat-sen University, Guangzhou, Guangdong, China; Louisiana State University Health Sciences Center, United States of America

## Abstract

Clonorchiasis, which is induced by the infection of *Clonorchis sinensis* (*C. sinensis*), is highly associated with cholangiocarcinoma. Because the available examination, treatment and interrupting transmission provide limited opportunities to prevent infection, it is urgent to develop integrated strategies to prevent and control clonorchiasis. Glycolytic enzymes are crucial molecules for trematode survival and have been targeted for drug development. Hexokinase of *C. sinensis* (*Cs*HK), the first key regulatory enzyme of the glycolytic pathway, was characterized in this study. The calculated molecular mass (Mr) of *Cs*HK was 50.0 kDa. The obtained recombinant *Cs*HK (r*Cs*HK) was a homotrimer with an Mr of approximately 164 kDa, as determined using native PAGE and gel filtration. The highest activity was obtained with 50 mM glycine-NaOH at pH 10 and 100 mM Tris-HCl at pH 8.5 and 10. The kinetics of r*Cs*HK has a moderate thermal stability. Compared to that of the corresponding negative control, the enzymatic activity was significantly inhibited by praziquantel (PZQ) and anti-r*Cs*HK serum. r*Cs*HK was homotropically and allosterically activated by its substrates, including glucose, mannose, fructose, and ATP. ADP exhibited mixed allosteric effect on r*Cs*HK with respect to ATP, while inorganic pyrophosphate (PPi) displayed net allosteric activation with various allosteric systems. Fructose behaved as a dose-dependent V activator with the substrate glucose. Glucose-6-phosphate (G6P) displayed net allosteric inhibition on r*Cs*HK with respect to ATP or glucose with various allosteric systems in a dose-independent manner. There were differences in both mRNA and protein levels of *Cs*HK among the life stages of adult worm, metacercaria, excysted metacercaria and egg of *C. sinensis*, suggesting different energy requirements during different development stages. Our study furthers the understanding of the biological functions of *Cs*HK and supports the need to screen for small molecule inhibitors of *Cs*HK to interfere with glycolysis in *C. sinensis*.

## Introduction

Human clonorchiasis is caused by infection with *Clonorchis sinensis* (*C. sinensis*), the Chinese or oriental liver fluke, which is mainly distributed in various eastern parts of Asia, including China, Korea, and Northern Vietnam. Approximately 35 million people suffer from infection with *C. sinensis* worldwide, of whom 15 million are Chinese [1]. Clonorchiasis is a tropical disease that was once neglected. Since it was first shown that *C. sinensis* infection was highly associated with cholangiocarcinoma [2]–[4], the World Health Organization (WHO) announced in 2009 that *C. sinensis* infection was one of the biological agents that could induce cholangiocarcinoma [5]. Because the available fecal examination, treatment of patients with praziquantel (PZQ) and interrupting transmission provide limited abilities to prevent the infection, it is urgent to develop integrated strategies to prevent and control clonorchiasis.

The genome and transcriptome data of *C. sinensis* demonstrate that the genes that are involved in both the glycolytic pathway and Krebs cycle are expressed during the infection [6], [7]. The *C. sinensis* adult transports external glucose in muscular tissues and breaks it down through glycolysis, producing lactic acid as the major end product, to supply energy and intermediate products for physiological metabolism [8], [9]. Glycolytic enzymes are crucial molecules for trematode survival and have been targeted for drug development [10], [11].

Hexokinase (ATP: D-hexose-6-phosphotransferase, EC 2.7.1.1.) is the first key regulatory enzyme of glycolytic pathway [12]. The product of catalysis, glucose-6-phosphate (G6P), also serves as a precursor for pentose phosphate pathway, which yields NADPH and pentose sugars [13], [14]. NADPH maintains the redox potential, and pentose sugars are used in the biosynthesis of nucleic acids [15]. Hexokinases (HKs) can be distinguished based on their molecular mass (Mr) and sensitivity to G6P inhibition. Generally, the Mr of HKs from non-mammalian organisms is approximately 50 kDa. Yeast hexokinase (yHK) is not inhibited by physiologically relevant levels of G6P [16], while HKs from various marine organisms, silkworm and *Schistosoma mansoni* (*S. mansoni*) show a reasonably potent inhibitory effect by G6P [17]–[19]. In mammals, three isozymes, HK-I, HK-II, and HK-III, have an Mr of approximately 100 kDa and exhibit marked sensitivity to inhibition by G6P [20]–[22]. These isozymes evolved by the duplication and fusion of a gene encoding an ancestral 50-kDa, G6P-sensitive HK [20], [22]–[25].

The key glycolytic enzymes in *C. sinensis* have been poorly studied compared to those of other parasites. HKs from *Leishmania mexicana* (*L. mexicana*) [26], *Brugia malayi* (*B. malayi*) [27], *Trypanosoma brucei* [28], [29], *Trypanosoma cruzi* (*T. cruzi*) [30]–[32], *Plasmodium falciparum* [33]–[35], *Haemonchus contortus* [36] and *S. mansoni* [19], [37], [38] are well-characterized targets for drug development. However, the characteristics of HK from *C. sinensis* (*Cs*HK) that allow for the design of a rational strategy of selective inhibition are unknown.

In this study, the sequence, structure, and enzymatic properties of *Cs*HK were analyzed. The molecular characteristics, including the Mr, mRNA and protein levels, during different life stages of *C. sinensis* were described. This enzyme is so important that the effects of pH, temperature, divalent cation, substrates and effectors on its enzymatic characteristics were extensively investigated. This study is a cornerstone for research on designing a rational strategy of selective inhibition of *Cs*HK to interfere with the glycolysis of *C. sinensis,* which may potentially prevent and treat clonorchiasis.

## Methods

### Ethics statement

BALB/c mice were purchased from the animal center of Sun Yat-sen University and were raised carefully in accordance with National Institutes of Health on animal care and the ethical guidelines. All of the experimental procedures were approved by the Animal Care and Use Committee of Sun Yat-sen University (Permit Numbers: SCXK (Guangdong) 2009-0011).

### Sequence analysis

The full-length cDNA sequence of *Cs*HK (Accession No. GAA52956.1) was downloaded from GenBank and analyzed using BLASTx provided by NCBI (http://www.ncbi.nlm.nih.gov/). The conserved domains and physico-chemical parameters of *Cs*HK were analyzed using bioinformatic tools that were provided by ExPASy (http://www.expasy.org/). The B-cell linear epitopes were analyzed by BepiPred (http://tools.immuneepitope.org/main/). The putative tertiary structure of *Cs*HK was constructed based on comparative modeling using SWISS-MODEL, viewed by Swiss-Pdb Viewer, and additionally evaluated with the Q-MEAN server [39], [40]. The phylogenetic tree of HKs was constructed using MEGA version 5.

### Purification of recombinant *Cs*HK (r*Cs*HK) and the preparation of mouse anti-r*Cs*HK serum

The forward primer (5′- GCCGAATTCATGGGATCCGTAGAAGAG-3′) containing an *EcoR* I restriction site (underlined) and the reverse primer (5′- ATTACTCGAGGCGACAAGATGCAGCG-3′) harboring the *Xho* I restriction site (underlined) were used to amplify open reading frame (ORF) of *Cs*HK from the total cDNA of adult *C. sinensis* via polymerase chain reaction (PCR). The reaction was carried out for 35 cycles at 95°C for 45 sec, 60°C for 45 sec, and 72°C for 1 min followed by an extension at 72°C for 10 min. The specific PCR products were purified, digested with *EcoR* and *Xho* and then subcloned into the prokaryotic expression vector pET-28a (+) (Novagen, Germany) that had been predigested with the same enzymes.

The recombinant plasmids were identified by digestion with *EcoR* and *Xho* and confirmed by DNA sequencing. The recombinant plasmids of pET-28a (+)-*Cs*HK were transformed into *E. coli* BL21 (DE3) (Promega, USA). After being induced by 1 mM IPTG (Sigma, USA) at 37°C for 5 h, the transformed cells were harvested by centrifugation for 15 min at 10,000×g at 4°C, suspended in lysis buffer (0.5 M NaCl, 20 mM Tris-HCl, and 5 mM imidazole, pH 8.0), sonicated on ice, and centrifuged at 10,000×g for 15 min at 4°C. r*Cs*HK was purified from the supernatant of the lysate using the His-bind purification kit (Novagen, USA) according to the manufacturer’s instructions. The purified samples were subjected to 12% sodium dodecyl sulfate polyacrylamide gel electrophoresis (SDS-PAGE), routinely processed for two-dimensional gel electrophoresis and confirmed by mass spectrometry (MS). Peptide mass spectra were obtained on an ABI 4800 Proteomics Analyzer MALDI-TOF/TOF (Applied Biosystems, USA). Both the MS and tandem mass spectrometry (MS/MS) data were interpreted and processed using the GPS Explorer software (version 3.6, Applied Biosystems, USA) to match the protein name.

The final concentration of *Cs*HK was determined using the bicinchoninic acid (BCA) protein assay kit (Novagen, USA). A 50-µg aliquot of r*Cs*HK was mixed with an equal volume of complete Freund’s adjuvant (Sigma, USA) and injected subcutaneously into a male BALB/c mouse (4-weeks-old). Then, the mouse was boosted with 25 µg of r*Cs*HK that was mixed with equivalent incomplete Freund’s adjuvant (Sigma, USA) 2 weeks and 4 weeks after the first injection. Anti-r*Cs*HK serum was collected 2 weeks after the last injection. Prior to the first injection, the sera were collected as naive sera.

### Determination of the apparent molecular mass (Mr) of r*Cs*HK

r*Cs*HK was subjected to 8% native PAGE. Thyroglobulin (669 kDa), ferritin (440 kDa), catalase (232 kDa), lactate dehydrogenase (140 kDa) and bovine serum albumin (67 kDa) (HMW Native, GE Healthcare, USA) were used as standard proteins. The HRm values of the molecular markers were plotted against their Mr. The equation and the curve were drawn. The Mr of r*Cs*HK was calculated using the obtained equation [41].

The Mr of r*Cs*HK was also determined using a calibration curve between the elution volume (Ve) and log Mr (kDa) of standard marker proteins by AKTA FPLC (GE Healthcare, USA) using a sepharose 12 10/300 GL (Tricorn, USA) gel filtration column [27]. The following gel filtration markers (Sigma-Aldrich, USA), viz. cytochrome c from equine heart (12.4 kDa), carbonic anhydrase from bovine erythrocytes (29 kDa), albumin from bovine serum (66 kDa), alcohol dehydrogenase from yeast (150 kDa), β-amylase from sweet potato (200 kDa), and blue dextran (2,000 kDa, for void volume (Vo) determination), were used to obtain the values of elution volume (Ve). The gel filtration column was run in 50 mM Tris-HCl (pH 7.5) containing 100 mM KCl at a flow rate of 0.8 ml/min. The Ve of r*Cs*HK was obtained, and the Mr of r*Cs*HK was calculated using the deduced equation.

### Western blotting analysis

Purified r*Cs*HK (2 µg) and total worm extract (30 µg) were subjected to 12% SDS-PAGE and then electrotransferred onto a polyvinylidene difluoride (PVDF) membrane (Whatman, UK) at 100 V for 1 h in a Trans-Blot transfer cell (Bio-Rad, USA). The PVDF membranes were blocked with 5% (w/v) skimmed milk in phosphate buffer saline (PBS, pH 7.4) overnight at 4°C and then probed with a mouse anti-His tag monoclonal antibody (1∶2,000 dilution, Novagen, USA), mouse anti-r*Cs*HK serum (1∶2,000 dilution) or serum from a pre-immune mouse (1∶2,000 dilution) for 2 h at room temperature. After washing with PBS 3 times, the membranes were incubated in horseradish peroxidase (HRP)-conjugated goat anti-mouse IgG (1∶2,000 dilution, Protein tech., USA) for 1 h at room temperature. Both the primary and secondary antibodies were diluted with 0.1% BSA in PBS (pH 7.4). After washing 5 times, the membranes were finally developed in color with diaminobenzidine (DAB, Boster, China) reagents according to the manufacturer’s instructions.

### Effects of pH, temperature and divalent cations on the enzyme kinetics of r*Cs*HK

A 200-µL aliquot of reaction system A included 3 mM glucose, 3 mM adenosine triphosphate (ATP, Sigma, USA), 15 mM MgCl_2_, 0.5 mM nicotinamide adenine dinucleotide phosphate (NADP, Sigma, USA), 0.3 U of yeast glucose 6-phosphate dehydrogenase (yG6PD) Type VII (Sigma, USA), and 100 mM Tris-Cl pH 8.5 [42]. Reduced nicotinamide adenine dinucleotide phosphate (NADPH) formation by glucose 6-phosphate (G6P) dehydrogenation was monitored at 340 nm in a microplate reader (SpectraMax M5, Molecular Devices, USA). To measure the optimal operating pH of r*Cs*HK, different buffers (50 mM glycine-NaOH and 100 mM Tris-Cl) were used to generate a pH range from 6.0 to 13.0. The optimum operating temperature of r*Cs*HK was determined at different temperatures (20–60°C) in the system. The thermal stability was measured by incubating the enzyme at 50°C. The samples (10 µL) were removed at different time points and immediately assayed as described above. The divalent cation requirement was assayed by variable concentrations of MgCl_2_, MnCl_2_, CaCl_2_, ZnCl_2_, or CuCl_2_ in the system.

In the reaction system A, effects of pH, temperature, and Mg^2+^ on the enzyme kinetics of G6PD, the reporter enzyme, were screened. Standard G6PD assay mixture containing 15 mM MgCl_2_, 0.5 mM NADP (Sigma, USA), 0.5 mM G6P (Sigma, USA), 0.3 U yG6PD Type VII, and 100 mM Tris-Cl pH 8.5, was monitored spectrophotometrically at 340 nm as previously described.

### Effects of praziquantel (PZQ) and anti-r*Cs*HK polyclonal antibody on r*Cs*HK

The effects of praziquantel (PZQ) on the enzymatic activity were also tested in this system [43], [44]. PZQ was dissolved in anhydrous ethanol and added to the system at final concentrations of 2–8 mM. A standard G6PD assay in the presence of different concentrations of PZQ was as a control reaction to determine the sensitivity of the reporter assay to PZQ. Equal volumes of anhydrous ethanol were added as controls. Mouse anti-r*Cs*HK serum was added to the system at dilutions of 1∶640-1∶5, and serum from a pre-immune mouse at the same dilutions was a negative control.

### Substrates kinetics and effectors of r*Cs*HK

To examine the phosphorylation of hexoses or the inhibitory effect of G6P on r*Cs*HK, the production of adenosine diphosphate (ADP) was assayed by coupled reactions of pyruvate kinase and lactate dehydrogenase by monitoring the decrease in NADH at 340 nm (ADP assay) in a microplate reader [42]. A 200-µL aliquot of reaction system B was composed of 3 mM of glucose, mannose or fructose, 3 mM ATP, 100 mM KCl, 15 mM MgCl_2_, 0.4 mM NADH, 5 mM phosphoenolpyruvate, 1 U of rabbit muscle pyruvate kinase (Sigma, USA), 2 U rabbit muscle lactate dehydrogenase (Sigma, USA), and 100 mM Tris-Cl (pH 8.5). To determine the kinetic parameters of r*Cs*HK, the substrate (glucose, mannose, fructose, or ATP) concentrations were varied from 0.05 to 2.5 mM in reaction system A and 0.05 to 3.0 mM in reaction system B. ADP (0–3 mM), inorganic pyrophosphate (PPi, 0–3 mM), and fructose (0–1 mM) were added to reaction system A, and G6P (0–4 mM) was added to reaction system B to investigate their effects on the enzymatic activity of r*Cs*HK.

The HK reaction was the rate-limiting step under every condition. All of the assays were carried out at in least three independent experiments that were performed in triplicate. The reactions were monitored for 5 min, and the initial velocity was calculated from a tangent that was fitted to the reaction curve. All of the kinetic datasets were fitted to the modified version of the Hill equation *v*  =  *V*_max_[S]^h^/(*K*_0.5_^h^ + [S]^h^) with OriginPro 8 SR0 (OriginLab Corporation, USA), where *V*_max_ represents the maximum velocity of the enzymatic reaction, [S] represents the concentration of the substrate, *K*_0.5_ represents the apparent affinity coefficient of the enzyme for its substrate, and h is the Hill coefficient defining the degree of cooperation of the allosteric enzymes [45]. One unit of HK activity is defined as the amount of the enzyme that catalyzes the production of 1 µmol G6P or ADP per min at 37°C.

### mRNA and protein levels of *Cs*HK during different life stages of *C. sinensis*

Adult worms, eggs, metacercaria, and excysted metacercaria were collected as previously described [46]. The total RNA was extracted from adult worms, eggs, metacercaria, or excysted metacercaria using TRIZOL (Invitrogen, USA) according to the manufacturer’s instructions and excluded the interference of DNA with DNase (Promega, USA). Real-time PCR was carried out using the primers 5′- CTATTGTAGAGCCGTTGGAT-3′ and 5′-TGATGTCGGTCATTCGTT-3′. β-actin of *C. sinensis* (GenBank accession No. EU109284) was used as an internal control [47], which was amplified with the primers 5′-ACCGTGAGAAGATGACGCAGA-3′ and 5′-GCCAAGTCCAAACGAAGAATT-3′. SYBR Premix Ex Taq Kit (TaKaRa, Japan) was used. A 20-µL aliquot of the PCR reaction mixture contained 2 µL of cDNA template, 10 µL of SYBR Premix Ex Taq buffer, 0.4 µL of the respective primers (10 µM), and 7.2 µL ddH_2_O. Real-time PCR was carried out in an iQ5 Real Time PCR Detection System (Bio-Rad, USA). The real-time PCR program was 95°C for 3 min, followed by 40 cycles of 95°C for 10 sec and 55°C for 30 sec. Finally, the melting curves were constructed using the following program: 95°C for 30 sec and 60°C for 15 sec, followed by increasing to 95°C while continuously collecting the fluorescence signal. A semiquantitative analysis was performed using the comparative 2^−ΔΔCt^ method [47], [48].

Western blotting was employed to investigate protein level of *Cs*HK during the life stages of adult worms, eggs, metacercaria, and excysted metacercaria. Parasites from these stages were respectively suspended in the RIPA lysis buffer (containing 1 mM proteinase inhibitor PMSF, Bioteke, China). The supernatant was collected after centrifugation for 15 min at 10,000× g at 4°C. The concentrations of total proteins were determined using BCA protein assay kit (Novagen, USA). 50 µg of total proteins from each life stage were separated on 12% SDS-PAGE and blotted onto PVDF membrane (Whatman, UK). The blots were developed with mouse anti-r*Cs*HK serum (1∶200 dilution), serum from pre-immune mouse (1∶200 dilution) and HRP-conjugated goat anti-mouse IgG (1∶2,000 dilution, Protein tech., USA). Detection was then carried out by chemiluminescence. The relative quantitation of protein levels were analyzed by Tanon Gis software (Tanon 4100, Shanghai, China).

### Statistical analysis

All of the experiments were repeated at least three times. SPSS version 13.0 software was used in this study for all of the statistical analyses. Student’s t test was used to analyze the measurement data among the groups. The results were represented as mean ± SD, and a *p* value<0.05 was classified as statistically significant.

## Results

### Sequence analysis of *Cs*HK

**Figure 1:**
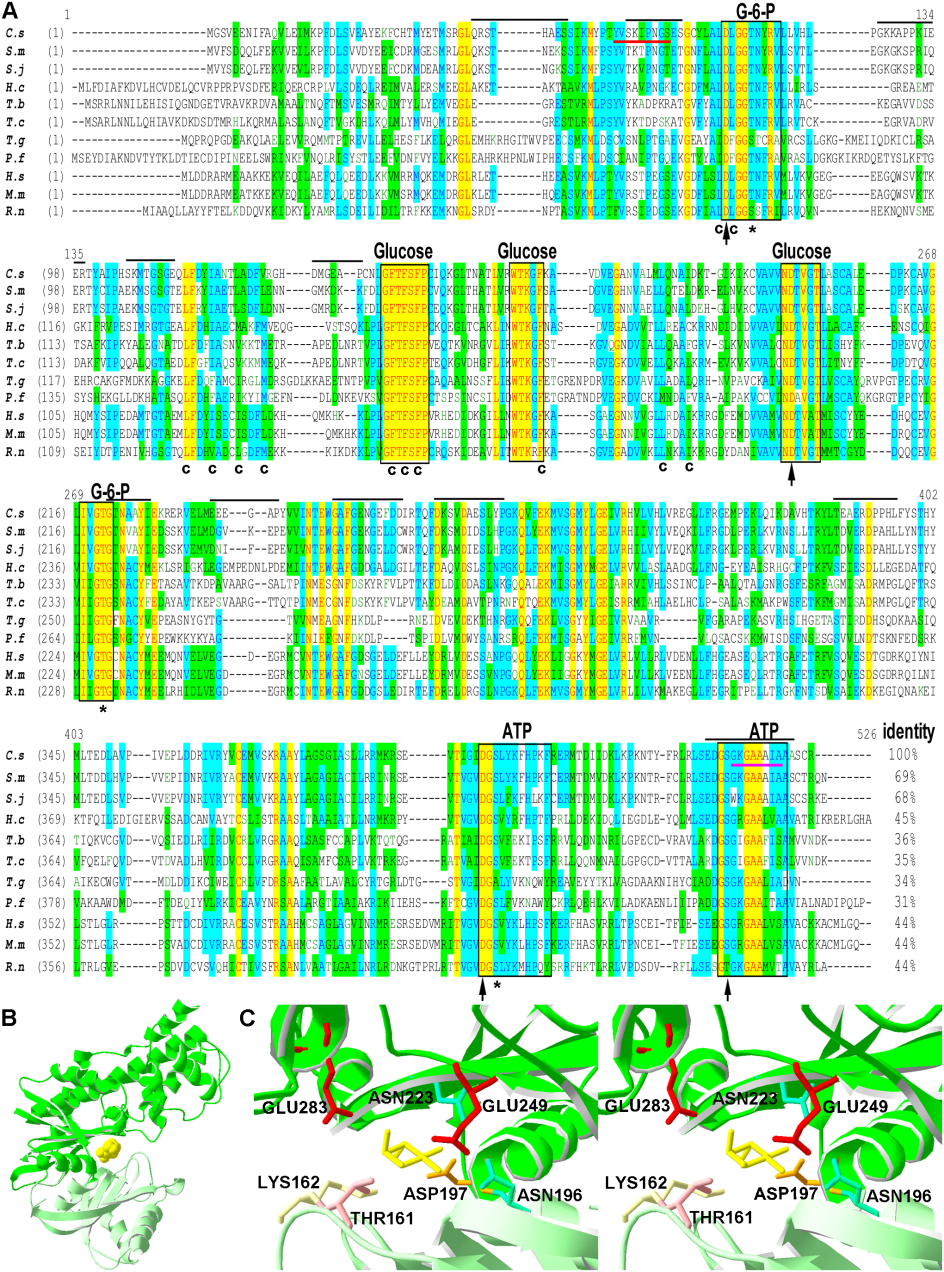
Sequence analysis and putative tertiary modeling of *Cs*HK. (A) Alignment of the amino acid sequence of *Cs*HK with that of HKs from other species. *Clonorchis sinensis* (*C. s*, GAA52956.1), *Schistosoma mansoni* (*S. m*, XP_002575647.1), *Schistosoma japonicum* (*S. j*, CAX74187.1), *Haemonchus contortus* (*H. c*, CAB40412.1), *Trypanosoma brucei* (*T. b*, CAC69958.1), *Trypanosoma cruzi* (*T. c*, AAL93565.1), *Toxoplasma gondii* (*T. g*, BAB55664.1), *Plasmodium falciparum* (*P. f,* AAA29613.1), *Homo sapiens* (*H. s*, NP_000153.1, *Mus musculus* (*M. m*, NP_034422.2), and *Rattus norvegicus* (*R. n*, NP_036866.1). The conserved domains for glucose, ATP and G6P were illustrated in the rectangles. The black arrows indicate the binding sites of the sugar moiety of G6P. The asterisks indicate the binding sites of the phosphate moiety of G6P. The residues that are marked “C” are present in the conserved hydrophobic channel. The 11 putative, linear B-cell epitopes with higher credibilities are marked with an overline. The putative connecting region I and α 13 helix region are respectively marked with red underline and magenta underline. (B) Ribbon drawing of the *Cs*HK putative structure model complexed with glucose (yellow, ball model). The spatial relationship of the large domain (green) and the small domain (light green) exhibit a closed form. The two domains are connected by connecting regions I–III. Glucose is shown as bound in the binding cleft of the domains. (C) Stereo view of the putative glucose-binding sites in *Cs*HK according to the molecular model of the HK from *S. mansoni* (PDB entry: 1BDG) [49]. Glucose (yellow stick) binds to the bottom of the deep cleft between the large domain (green) and the small domain (light green). The glucose-binding sites, including ASP197 (brown stick), ASN223 (light blue stick), GLU249 (red stick), GLU283 of the large domain, THR161 (pink stick), LYS162 (light yellow stick) of the small domain, and ASN196 of connecting region II (light green), are indicated.

The ORF of *Cs*HK consisted of 1350 bp encoding 449 amino acids. BLASTx showed that the deduced amino acid sequence of *Cs*HK, respectively, shared 69%, 68%, 45%, 36%, 35%, 34%, 31%, 44%, 44% and 44% identity with HK from *Schistosoma mansoni* (*S. mansoni*, XP_002575647.1, hexokinase), *Schistosoma japonicum* (*S. japonicum*, CAX74187.1, hexokinase A), *Haemonchus contortus* (*H. contortus*, CAB40412.1, hexokinase), *Trypanosoma brucei* (*T. brucei*, CAC69958.1, hexokinase), *Trypanosoma cruzi* (*T. cruzi*, AAL93565.1, hexokinase), *Toxoplasma gondii* (*T. gondii*, AAL93565.1, hexokinase), *Plasmodium falciparum* (*P*. *falciparum,* AAA29613.1, hexokinase type IV), *Homo sapiens* (*H. sapiens*, NP_000153.1, glucokinase isoform 1), *Rattus norvegicus* (*R. norvegicus*, NP_036866.1, the N-terminal half of rat hexokinase-1) and *Mus musculus* (*M. musculus*, NP_034422.2, glucokinase) (Figure 1A). As in HKs from other species, there were predicted to be multiple binding sites of glucose, ATP, sugar moiety of G6P and phosphate moiety of G6P in the amino acid sequence of *Cs*HK as well as a conserved hydrophobic channel [49], [50]. There were 20 putative B-cell linear epitopes in the amino acid sequence of *Cs*HK.

A three-dimensional structure of *Cs*HK was constructed according to the molecular model of HK from *S. mansoni* (PDB entry: 1BDG) by SWISS-MODEL, which shared 69% identity with *Cs*HK. A QMEAN4 score of 0.670 and a z-score of −1.554 were reported, supporting the quality of the model. *Cs*HK was composed of a large domain (green) and a small domain (light green). There were connecting regions I-III between the two domains (Figure 1B). A stereo view of putative glucose binding sites in *Cs*HK was shown according to the molecular model of HK from *S. mansoni* (Figure 1C) [49]. Glucose (yellow stick model) bound to the bottom of the deep cleft between the large domain (green) and the small domain (light green).

**Figure 2:**
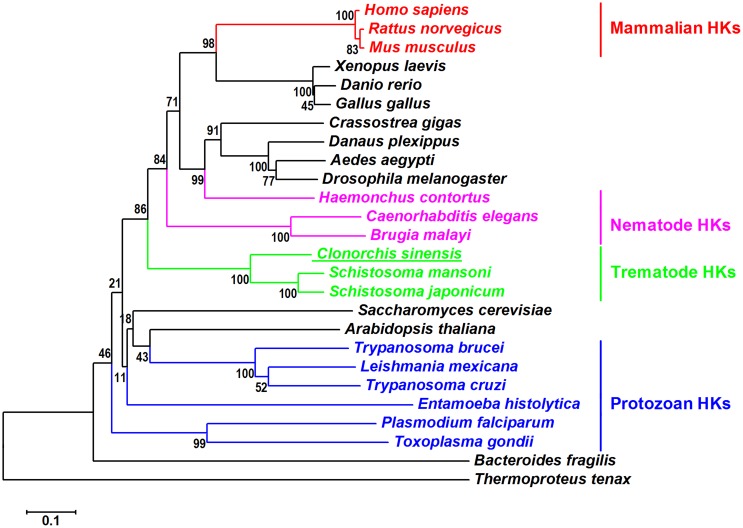
Neighbor-joining phylogenetic tree of HKs. The bootstrap values are shown at the branching point (1,000 replications). The amino acid sequences were retrieved from DDBJ and GenBank. The sequences are as follows: *Homo sapiens* (NP_000153.1, glucokinase isoform 1), *Rattus norvegicus* (P17712.2, Glucokinase), *Mus musculus* (NP_034422.2, glucokinase), *Xenopus laevis* (AAH72832.1, Hk1-A protein), *Danio rerio* (AAH67330.1, Hexokinase 1), *Gallus gallus* (BAC20932.1, hexokinase1), *Crassostrea gigas* (EKC42666.1, Hexokinase type 2), *Danaus plexippus* (EHJ75730.1, hexokinase), *Aedes aegypti* (EAT38749.1, hexokinase), *Drosophila melanogaster* (AGB95227.1, hexokinase A, isoform C), *Haemonchus contortus* (CAB40412.1, hexokinase), *Caenorhabditis elegans* (NP_492905.1, Protein H25P06.1), *Brugia malayi* (AAR13363.1, hexokinase), *Clonorchis sinensis* (GAA52956.1, hexokinase), *Schistosoma mansoni* (XP_002575647.1, hexokinase), *Schistosoma japonicum* (CAX74187.1, Hexokinase A), *Saccharomyces cerevisiae* (AAA34699.1, hexokinase (HXK2)), *Arabidopsis thaliana* (Q42525.2, hexokinase-I), *Trypanosoma brucei* (CAC69958.1, hexokinase), *Leishmania mexicana* (XP_003875265.1, putative hexokinase), *Trypanosoma cruzi* (AAL93565.1, hexokinase), *Entamoeba histolytica* (CAA57682.1, hexokinase), *Plasmodium falciparum* (AAA29613.1, hexokinase type IV), *Toxoplasma gondii* (BAB55664.1, hexokinase), *Bacteroides fragilis* (AAT74901.1, general specificity hexokinase), and *Thermoproteus tenax* (CAD52839.1, hexokinase).

As shown in the phylogenetic tree of HKs (Figure 2), *Cs*HK was most closely related to the HKs from *S. mansoni* and *S. japonicum*. *Cs*HK was also closely related to the HKs from nematodes, followed by vertebrates, but was distantly related to the HKs form protozoa.

### Expression and purification of r*Cs*HK

The putative pI of *Cs*HK was 6.17. The instability index (II) in *E. coli* was predicted to be 34.39, suggesting that the protein was stable. There was no transmembrane region or subcellular localization sequences (data not shown). The soluble r*Cs*HK was expressed with a 6× His-tag in *E. coli* B21. The purified recombinant protein showed a single band of approximately 54.8 kDa (containing His-tag), which was consistent with the predicted Mr (Figure S1). The peptides in the purified protein that were identified by MS assay were matched to those of *Cs*HK (Figure S2). The final protein concentration of r*Cs*HK was as high as 1.3 mg/ml. In terms of yield, 9.0–13 mg of r*Cs*HK could be reproducibly obtained from each fermentation batch comprising of 1 L of *E. coli* culture.

### Apparent molecular mass (Mr) of r*Cs*HK

**Figure 3:**
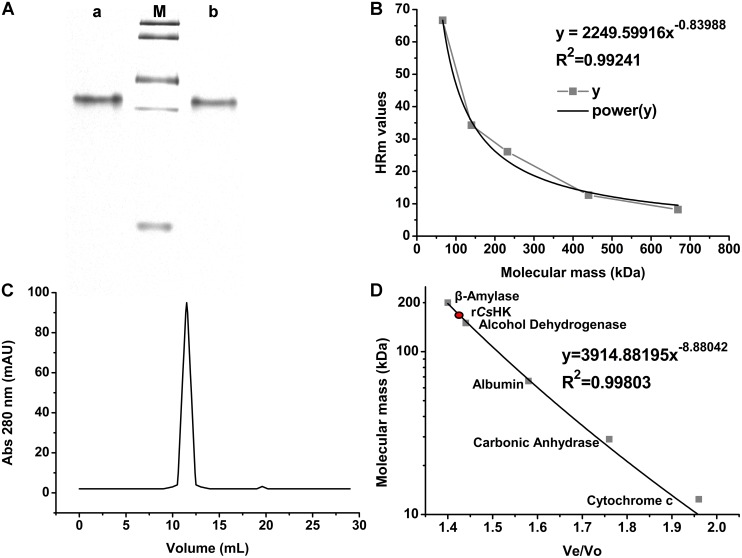
Determination of the Mr of r*Cs*HK. (A). M, the protein bands with known molecular mass (HMW Native, GE Healthcare, USA) were stained with Coomassie blue in descending order: thyroglobulin (669 kDa), ferritin (440 kDa), catalase (232 kDa), lactate dehydrogenase (140 kDa), and bovine serum albumin (67 kDa). a, Fresh purified r*Cs*HK. b, r*Cs*HK stored for 4 weeks at −80°C with four cycles of freezing and thawing. (B). The Mr of r*Cs*HK as determined by 8% native PAGE. The HRm values of the molecular markers were plotted against their Mr. The equation and curve were drawn. The Mr of r*Cs*HK was calculated using the obtained equation. (C). Elution profile of r*Cs*HK in gel filtration chromatography. (D). Mr of r*Cs*HK as determined by the elution profile. The calibration curve between the elution volume (Ve) and the log molecular mass (kDa) of standard marker proteins was obtained by AKTA FPLC using a sepharose 12 10/300 GL gel filtration column. The Ve of r*Cs*HK was obtained, and the Mr of r*Cs*HK was calculated using the deduced equation.

The mobility of r*Cs*HK was between that of catalase (232 kDa) and that of lactate dehydrogenase (140 kDa). The purified protein was stable after four cycles of freezing and thawing (Figure 3A). Plotting the HRm against the Mr of the markers produced an equation describing the electrophoretic profile. Based on this equation, the Mr of r*Cs*HK was calculated to be approximately 164 kDa (Figure 3B). In gel filtration chromatography, r*Cs*HK was eluted as a single peak between alcohol dehydrogenase (150 kDa) and β-amylase (200 kDa) (Figure 3C and D).

### Western blotting analysis

**Figure 4:**
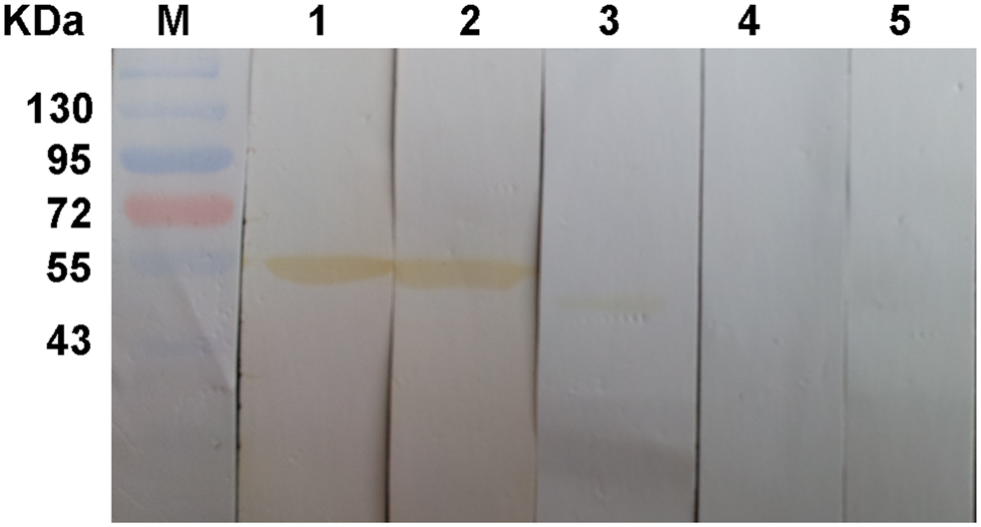
Western blotting analysis of r*Cs*HK. Pre-stained protein markers (lane M), r*Cs*HK reacted with the mouse anti-His tag monoclonal antibody (lane 1), r*Cs*HK probed with the mouse anti-r*Cs*HK serum (lane 2), total worm extract blotted with the mouse anti-r*Cs*HK serum (lane 3), r*Cs*HK probed with the naive mouse serum (lane 4), and total worm extract reacted with the serum from the pre-immune mouse (lane 5).

r*Cs*HK was probed with a mouse anti-His tag monoclonal antibody and mouse anti-r*Cs*HK serum yielding a cross-reactive band of approximately 54.8 kDa while not recognized by naive mouse serum. In addition, total worm extract was blotted with anti-r*Cs*HK mouse serum of approximately 50.0 kDa but not with serum from a pre-immune mouse (Figure 4).

### Effects of pH, temperature and divalent cations on the enzyme kinetics of r*Cs*HK

**Figure 5:**
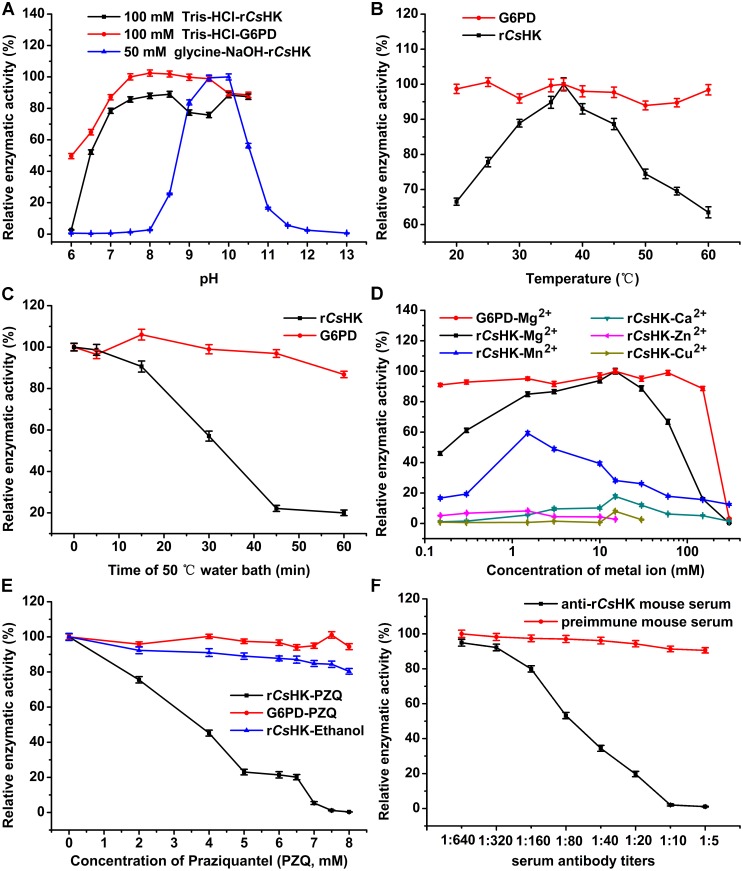
Enzyme activity analysis of r*Cs*HK in reaction system A. (A) Optimal pH analysis for r*Cs*HK. Different buffers were used to generate a pH range from 6.0 to 13.0 (50 mM glycine-NaOH and 100 mM Tris-Cl). (B) Enzymatic activity of r*Cs*HK at 20°C–60°C. (C) Thermal stability of the enzyme measured at time points at 50°C. (D) The effects of divalent cations (Mg^2+^, Mn^2+^, Ca^2+^, Zn^2+^, or Cu^2+^) on the enzymatic activity of r*Cs*HK. (E) The effect of praziquantel (PZQ). Equal volume of anhydrous ethanol as a control. (F) The effect of mouse anti-r*Cs*HK serum. The serum from a pre-immune mouse was used as a negative control. The dilutions of the sera ranged from 1∶640 to 1∶5.

The highest activity of r*Cs*HK was obtained with 100 mM Tris-HCl at pH 8.5 and 10, while in the 50 mM glycine-NaOH buffer, the peak activity was recorded at pH 10. In 100 mM Tris-HCl, more than 87% of the maximal activity of G6PD was observed between pH 7 and 10.5, and G6PD retained a relative enzymatic activity of 65% for pH 6.5 and 50% for pH 6 (Figure 5A). The optimal temperature for enzymatic activity of r*Cs*HK was 37°C. Between 20°C and 60°C, more than 60% of the maximal activity of r*Cs*HK and more than 94% of the maximal activity of G6PD were observed (Figure 5B). At 50°C, r*Cs*HK retained a relative enzymatic activity of 57% for 30 min and 21% for 1 hour, and G6PD retained a relative enzymatic activity of 97% for 45 min and 87% for 1 hour (Figure 5C). The kinetics of G6PD has a fine thermal stability. The enzyme was stable when stored for 2 weeks at 4°C (more than 90% of the activity was recovered), but very little enzyme activity was observed with one cycle of freezing and thawing (data not shown). The enzyme activity of r*Cs*HK decreased in the presence of different divalent cations on the order of Mg^2+^, Mn^2+^, Ca^2+^, Zn^2+^, and Cu^2+^. Mg^2+^ caused 84% inhibition at 150 mM, while 15 mM Mg^2+^ appeared to be an optimal concentration for enzyme activity. In the absence of Mg^2+^, no enzyme activity was detected. In the absence of Mg^2+^, G6PD showed 88% relative enzymatic activity. With 0.15–60 mM of Mg^2+^, more than 91% of the activity was recovered, while higher concentrations of Mg^2+^ had a significantly reduced relative enzymatic activity of 88% at 150 mM and 3% at 300 mM (Figure 5D).

### Effects of praziquantel (PZQ) and anti-r*Cs*HK polyclonal antibody on r*Cs*HK

Compared to equal volumes of anhydrous ethanol, which showed a slight inhibitory effect, the enzyme that was incubated with PZQ had a significantly reduced relative enzymatic activity of 45% at 4 mM and 0.3% at 8 mM. Relative enzymatic activity of G6PD was around 100% in the presence of 0–8 mM PZQ (Figure 5E). Compared to serum from the pre-immune mouse, which showed a slight inhibitory effect, the enzyme that was incubated with mouse anti-r*Cs*HK serum had a significantly reduced relative enzymatic activity of 53% at a 1∶80 dilution and 1% at a 1∶10 dilution (Figure 5F).

### Substrate kinetics and effectors of r*Cs*HK

**Figure 6:**
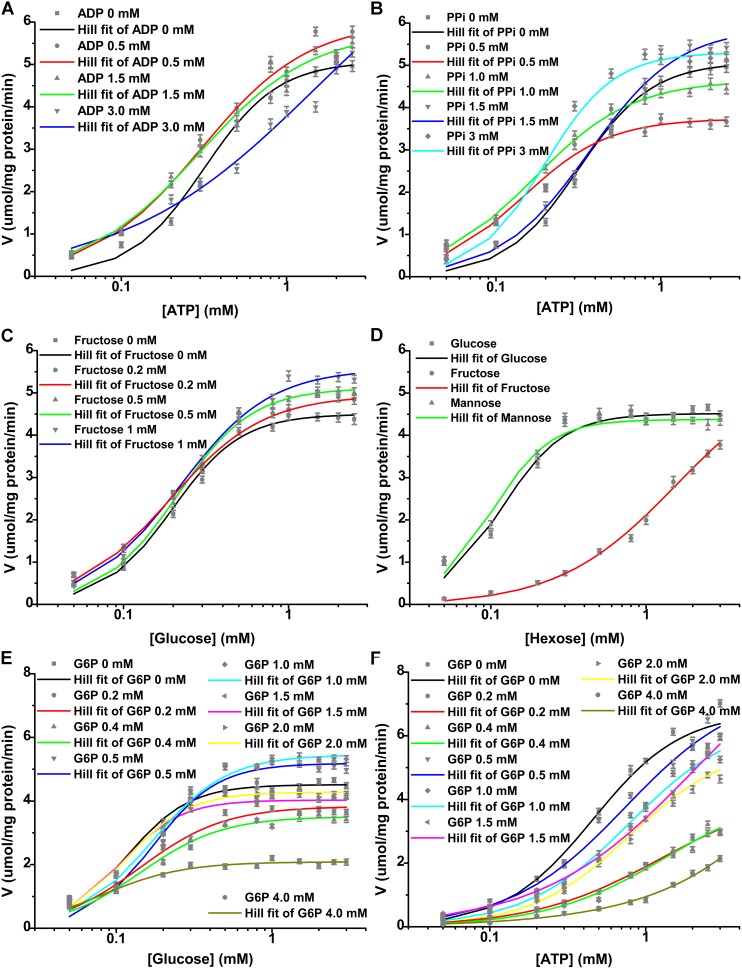
Enzymatic activity of r*Cs*HK with respect to different substrates or effectors. (A) The effect of 0∼3.0 mM ADP and fixed 3.0 mM glucose with respect to ATP in reaction system A. (B) The effect of 0∼3.0 mM PPi and fixed 3.0 mM glucose with respect to ATP in reaction system A. (C) The effect of 0∼1.0 mM fructose and fixed 3.0 mM ATP with respect to glucose in reaction system A. (D) The effect of 0∼3.0 mM hexoses (glucose, fructose and mannose) and fixed 3.0 mM ATP in reaction system B. (E) The effect of 0∼4.0 mM G6P and fixed 3.0 mM ATP with respect to glucose in reaction system B. (F) The effect of 0∼4.0 mM G6P and fixed 3.0 mM glucose with respect to ATP in reaction system B.

The substrate saturation curves for the HK substrates, including glucose, mannose, fructose, and ATP, were sigmoidal, which is characteristic of an allosteric enzyme (Figure 6). The *K*_0.5_ values (mM) for glucose, mannose, fructose, and ATP were 0.116±0.010, 0.101±0.009, 1.560±0.071 and 0.459±0.007, respectively (Table S1). r*Cs*HK was inhibited by high concentrations of ATP but not by high concentrations of glucose. At a physiological concentration (5 mM), ATP showed 8% inhibition of r*Cs*HK, while higher concentrations (50 mM) ATP showed 84% inhibition (Figure S3). r*Cs*HK did not present activity with PPi or ADP as substrates.

The allosteric site ligand ADP, which is a product of HK, exhibited an unusual mixed allosteric effect on *Cs*HK with respect to ATP. At lower concentrations, such as 0.5 mM and 1.5 mM, ADP behaved as a V activator [51], while at higher concentrations (3.0 mM), ADP behaved as a V activator and a K inhibitor (antiergistic or crossed mixed K−V+ effect [52]) (Figure 6A, Table S1). Under these conditions, V activation contributed less to the effective reaction rate, while K inhibition predominated. The resulting effect was a net inhibition by 3.0 mM ADP, with the reduction of h form 1.935±0.271 to 0.740±0.143.

Different concentrations of PPi displayed a net allosteric activation of r*Cs*HK with respect to ATP with various allosteric systems. Approximately 0.2∼1.0 mM fructose behaved as a dose-dependent V activator of r*Cs*HK with respect to glucose. The affinity of the enzyme for its substrate as expressed by *K*_0.5_ remained constant at different fructose concentrations, while the h and *V*_max_ values varied. The *V*_max_ increased with increasing concentrations of fructose (Table S1), which is characteristic of a V-type allosteric effector [51]. Different concentrations G6P displayed the net allosteric inhibition of r*Cs*HK with respect to ATP or glucose with various allosteric systems. The allosteric inhibition by G6P of r*Cs*HK was dose-independent. In addition, the allosteric inhibition by G6P of r*Cs*HK with respect to ATP was more significant than with respect to glucose.

The kinetics of r*Cs*HK in the absence and presence of allosteric effectors are shown in Figure 6. The parameters that were derived from these kinetics are summarized in Table S1.

### mRNA and protein levels of *Cs*HK at life stages of *C. sinensis*

**Figure 7:**
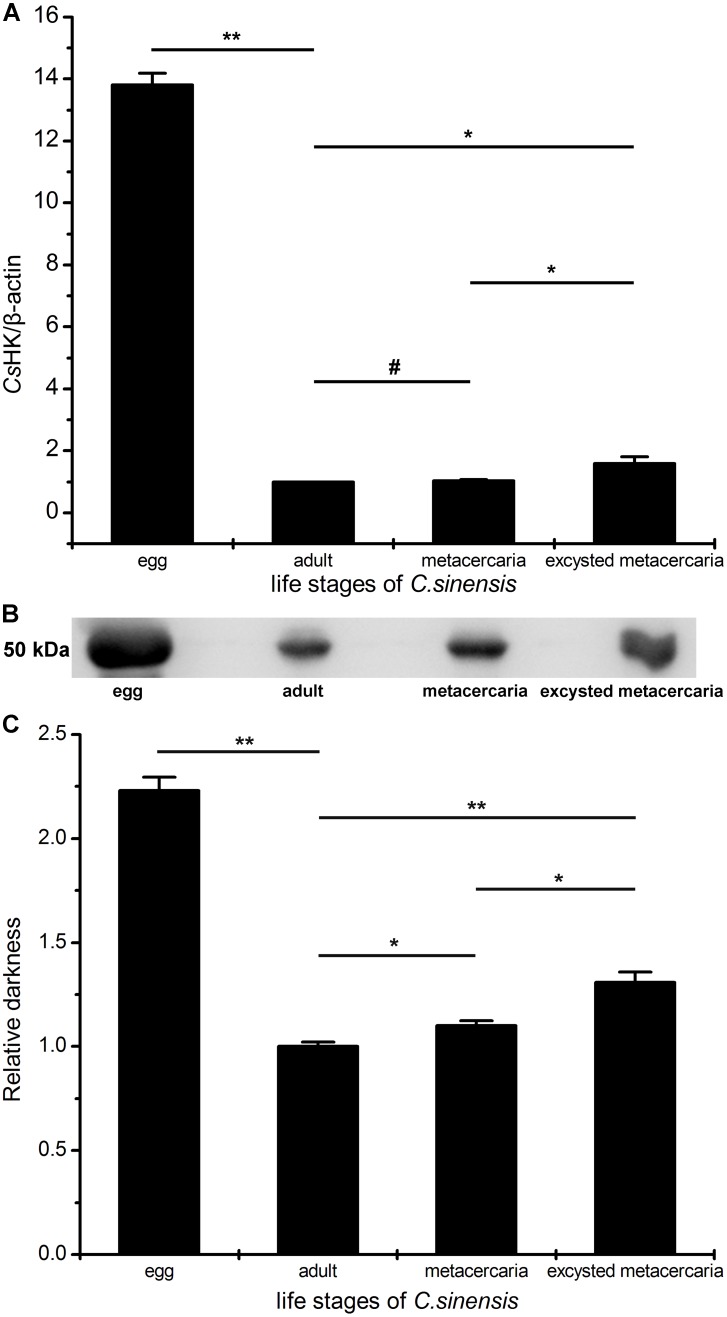
mRNA and protein levels of *Cs*HK at life stages of *C. sinensis*. (A) Real-time PCR analysis. The mRNA of β-actin of *C. sinensis* was used as an internal control. Semiquantitative analysis as performed by the comparative 2^−ΔΔCt^ method. There was no significant difference between the adult worm and metacercaria (*p*>0.05). There were statistical differences in the mRNA levels of *Cs*HK not only between adult worm and encysted metacercaria (*p*<0.05) but also between metacercaria and encysted metacercaria (*p*<0.05). The mRNA level of *Cs*HK in the egg was higher than that in the adult worm (13.80-fold, *p*<0.01), metacercaria (13.29-fold, *p*<0.01) and excysted metacercaria (8.72-fold, *p*<0.01). (B) Western blotting analysis. 50 µg of total proteins of each stage were blotted with mouse anti-r*Cs*HK serum (1∶200 dilution). Specific protein bands around 50 kDa were probed. There was no corresponding band in negative lanes (not shown). (C) The relative quantitation of protein levels were analyzed by Tanon Gis software. The protein level of *Cs*HK in the egg was the highest, followed by those of excysted metacercaria, metacercaria, and adult worm. The protein levels were consistent with the mRNA levels. The experiments were repeated three times. (#, *p*>0.05; *, *p*<0.05; **, *p*<0.01).

*Cs*HK mRNA can be detected during the stages of egg, adult worm, metacercaria, and excysted metacercaria of *C. sinensis* (Figure 7A). There was no significant difference in the mRNA levels of *Cs*HK between adult worms and metacercaria (*p*>0.05). There were statistical differences in the mRNA levels of *Cs*HK not only between adult worm and encysted metacercaria (*p*<0.05) but also between metacercaria and encysted metacercaria (*p*<0.05). The mRNA level of *Cs*HK in the egg was higher than that in the adult worm (13.80-fold, *p*<0.01), metacercaria (13.29-fold, *p*<0.01) and excysted metacercaria (8.72-fold, *p*<0.01).

Specific blots around 50 kDa were observed with mouse anti-r*Cs*HK serum by western blotting assay. The protein level of *Cs*HK in the egg was the highest, followed by those of excysted metacercaria, metacercaria, and adult worm (Figure 7B and C). The same dilution of pre-immune mouse serum was used as a negative control, and no corresponding bands were observed (not shown).

## Discussion

In this study, the sequence, structure, and enzymatic activity characteristics of *Cs*HK were investigated. An analysis of the amino acid sequence and spatial structure showed that *Cs*HK possesses typical characteristics of non-mammalian HK (Figure 1). A sequence alignment demonstrated that HKs represent a family sharing common evolutionary relationships [53] (Figure 2). Peptides of purified r*Cs*HK were confirmed by MS (Figure S2). When subjected to SDS-PAGE, the Mr of r*Cs*HK was approximately 54.8 kDa (containing His-tag) (Figure S1). r*Cs*HK was probed with a mouse anti-His tag monoclonal antibody of approximately 54.8 kDa (Figure 4), while the Mr of r*Cs*HK was approximately 164 kDa, as determined by native PAGE and gel filtration (Figure 3). The quantitative evaluation of the Hill coefficient (h) for substrate binding indicated that the enzyme was a polymer that was made up of subunits. These results indicate that *Cs*HK was a homotrimer, whereas the HKs from other species are monomers, dimers or tetramers [19], [20], [27], [30].

Considering that G6PD catalyzed the coupled reaction in an Mg^2+^-independent manner at range of 0–150 mM, r*Cs*HK catalyzed the conversion of hexose to hexose phosphate in an Mg^2+^-dependent manner (Figure 5D). The concentration of 15 mM MgCl_2_ appeared to be optimal for the enzyme activity because in the absence of Mg^2+^, no enzyme activity was detected, indicating that Mg-ATP is the true substrate.

Moreover, the highest activity was obtained with 100 mM Tris-HCl at pH 8.5 and 10, while in 50 mM glycine-NaOH buffer, the peak activity was recorded at pH 10 (Figure 5A). Our results indicate the adaptability of r*Cs*HK in the alkaline environment of biliary ducts. The kinetic behavior of the enzyme under different pH conditions suggests that the enzyme might undergo subtle conformational changes in response to the buffers [54].

PZQ has been successfully used to treat the majority of human-infecting trematodes and cestodes. The exact mechanism of action of PZQ against trematodes and cestodes remains unclear, although there are some theories [55], [56]. At 0.2 mM of PZQ, enzymatic activities of recombinant lactate dehydrogenase from *S. japonicum* (r*Sj*LDH) were inhibited by 83.8% for reduction reaction and 72.2% for oxidation reaction respectively [57]. Compared with the controls, the inhibition rate of 9 mM PZQ on recombinant lactate dehydrogenase from *C. sinensis* (r*Cs*LDH) catalyzing the oxidation-reduction reaction was 94% [44]. In our coupled reaction, PZQ was found to be ineffective for inhibition of G6PD, so that the relative enzymatic activity of r*Cs*HK reduced significantly by adding millimoles level of PZQ. The result provided a new viewpoint to investigate the mechanism of PZQ. It was suggested that the key enzymes of glycolysis, including *Cs*LDH and *Cs*HK, might be targets of PZQ against *C. sinensis*. In the further study, we will explore the effect of PZQ on native *Cs*HK and gather more evidence to know if *Cs*HK inhibition by PZQ has any relevance in the worm.

The enzyme that was incubated with mouse anti-r*Cs*HK serum had a significantly reduced relative enzymatic activity (Figure 5F). Mouse anti-r*Cs*HK serum was induced by putative B-cell epitopes in the amino acid sequence of *Cs*HK, including an ATP-binding site, connecting region I and α 13 helix region, which were respectively marked with rectangle, red underline and magenta underline in Figure 1. The sites are also necessary for the enzymatic activity [58], [59]. The characteristic might contribute to that anti-r*Cs*HK serum dominantly reduced the enzymatic activity of r*Cs*HK.

r*Cs*HK was activated by its substrates, including glucose, mannose, fructose, and ATP (Figure 6), in a comparable cooperation manner when expressed by Hill coefficients (h), suggesting the homotropic allosteric activation of the enzyme by its substrate. However, HKs of *L. mexicana* [26] and *T. gondii* [42] are not allosteric enzymes. Similar to HKs of *S. mansoni* [19], *B. malayi* [27] and *T. gondii* [42], *Cs*HK could use glucose, fructose, and mannose as substrates. The h, *K*_0.5_, *V*_max_ and *k*_cat_ values for glucose and mannose were similar and different from those of fructose (Table S1), indicating that r*Cs*HK had a low affinity for fructose and preferred glucose as well as mannose. r*Cs*HK did not present activity with PPi or ADP as substrates.

The allosteric site ligand, ADP, which is a product of HK, had mixed allosteric effect on r*Cs*HK with respect to ATP (Figure 6A, Table S1). r*Cs*HK was inhibited by high concentrations of ATP (Figure S3). The results suggest a major role of the ATP/ADP ratio in regulating the enzymatic activity of r*Cs*HK.

The HKs of *B. malayi* [27] and *T. cruzi* [30] were of mixed-type inhibited by PPi. The inhibition by PPi of the HK of *L. mexicana* was clearly competitive [26]. Different concentrations of PPi displayed the net allosteric activation of r*Cs*HK with respect to ATP with various allosteric systems (Figure 6B, Table S1). The physiological significance of the allosteric modulation by PPi was not clear and deserves a more-detailed investigation.

r*Cs*HK could use fructose, and the *K*_0.5_ value for fructose was 1.560±0.071 mM. Approximately 0.2∼1.0 mM fructose behaved as a dose-dependent V activator of r*Cs*HK with respect to glucose (Figure 6C, Table S1). The HK of *L. mexicana* could also use fructose, and the Km value for fructose (0.142 mM) is lower than that for fructose of nonspecific HKs from other sources. The enzyme was inhibited by fructose with a Ki = 0.230 mM with respect to glucose [26]. The HKs from *C. sinensis* and *L. mexicana* had considerably different affinities for fructose. With respect to glucose, fructose played a reversed role in r*Cs*HK and the HK from *L. mexicana.*

The inhibition by G6P has been studied in the 100-kDa type HKs and a few 50-kDa type enzymes. For instance, the HK of *L. mexicana* exhibits a moderate sensitivity to inhibition (competitive vs. ATP and glucose) by the product G6P [26]. G6P is a competitive inhibitor vs. ATP of the HK from *S. mansoni* [19], [38]. The HK of *B. malayi* is activated by G6P [27]. G6P and inorganic phosphate compete as inhibitors with ATP for a common anion-binding site. However, these residues are highly conserved, even in G6P-insensitive HKs, such as yHK. Subtle structural differences would give rise to more G6P sensitivity than simply local side chain substitutions [49]. r*Cs*HK is a 50-kDa G6P-sensitive allosteric modulation HK.

The 100-kDa HK-I, HK-II, and HK-III of mammalian hosts have high affinities for glucose (Km = between 7 and 200 µM) and are strongly inhibited by G6P. The 50-kDa HK-IV (HK-D), also called glucokinase, has a low affinity for glucose (Km = 5–12 mM) and is not regulated by G6P [16], [20], [60]. *Cs*HK is an allosteric enzyme that is different from the HKs of *L. mexicana* [26] and *T. gondii* [42]. The crystal structure and structural basis of the allosteric regulation of r*Cs*HK can be further clarified through the stability and availability of the purified protein. Collectively, *Cs*HK seems to share some characteristics with the HKs of mammals. These results contribute to a better understanding of the differences between *Cs*HK and the homologues from the host, allowing us to propose *Cs*HK as a potential target for the design of specific drugs against *C. sinensis*.

In addition, there were differences in both mRNA and protein levels of *Cs*HK among the life stages of adult worm, metacercaria, excysted metacercaria and egg of *C. sinensis* (Figure 7), suggesting different energy requirements during different development stages. The protein levels were consistent with the mRNA levels. Both mRNA and protein levels of *Cs*HK in the egg was the highest. In this stage, a large amount of energy is required for cell growth, differentiation, and proliferation, which might interpret the highest expression level.

In conclusion, *Cs*HK possesses typical characteristics of non-mammalian HKs regarding the amino acid sequence and spatial structure. r*Cs*HK is a homotrimer and a distinct 50-kDa G6P-sensitive allosteric HK. Our study is a cornerstone for research seeking to identify additional biological functions of *Cs*HK and designing a rational strategy of small molecule inhibitors of *Cs*HK to interfere with the glycolysis of *C. sinensis*.

## Supporting Information

Figure S1 **Expression and purification of r** ***Cs*** **HK by 12% SDS-PAGE.** r*Cs*HK was expressed in *E. coli* with IPTG induction and was purified by His-band resin chromatography. Protein markers (lane M), lysate of *E. coli* with pET-28a (+) without induction (lane 1) and with induction (lane 2), lysate of *E. coli* with pET-28a (+)-*Cs*HK without induction (lane 3) and with induction (lane 4), supernatant (lane 5) and precipitant (lane 6) of lysate of *E. coli* with pET-28a (+)-*Cs*HK with induction, and the purified recombinant *Cs*HK (lane 7).
(DOC)

Figure S2 **Identification of r** ***Cs*** **HK by MS.** The peptide mass spectra of the purified recombinant protein were obtained on an ABI 4800 Proteomics Analyzer MALDI-TOF/TOF, interpreted and processed using the GPS Explorer software. The identified peptides were matched to those of the hexokinase of *C. sinensis* [gi|358253389] in the NCBI database.
(DOC)

Figure S3 **The substrate saturation curves for r** ***Cs*** **HK substrates, including ATP and glucose.**
(DOC)

Table S1 **Summarized kinetic parameters of r** ***Cs*** **HK fitting the Hill equation.**
(XLS)
