# Supplementary material for: Sequence Analysis and Molecular Characterization of Clonorchis sinensis Hexokinase, an Unusual Trimeric 50-kDa Glucose-6-Phosphate-Sensitive Allosteric Enzyme
Source: PLoS One. 2014 Sep 18;9(9):e107940. doi: 10.1371/journal.pone.0107940 (PMC4169440; doi:10.1371/journal.pone.0107940)
Supplement: Figure S1 — Expression and purification of r Cs HK by 12% SDS-PAGE. rCsHK was expressed in E. coli with IPTG induction and was purified by His-band resin chromatography. Protein markers (lane M), lysate of E. coli with pET-28a (+) without induction (lane 1) and with induction (lane 2), lysate of E. coli with pET-28a (+)-CsHK without induction (lane 3) and with induction (lane 4), supernatant (lane 5) and precipitant (lane 6) of lysate of E. coli with pET-28a (+)-CsHK with induction, and the purified recombinant CsHK (lane 7). (DOC) [file pone.0107940.s001.doc]

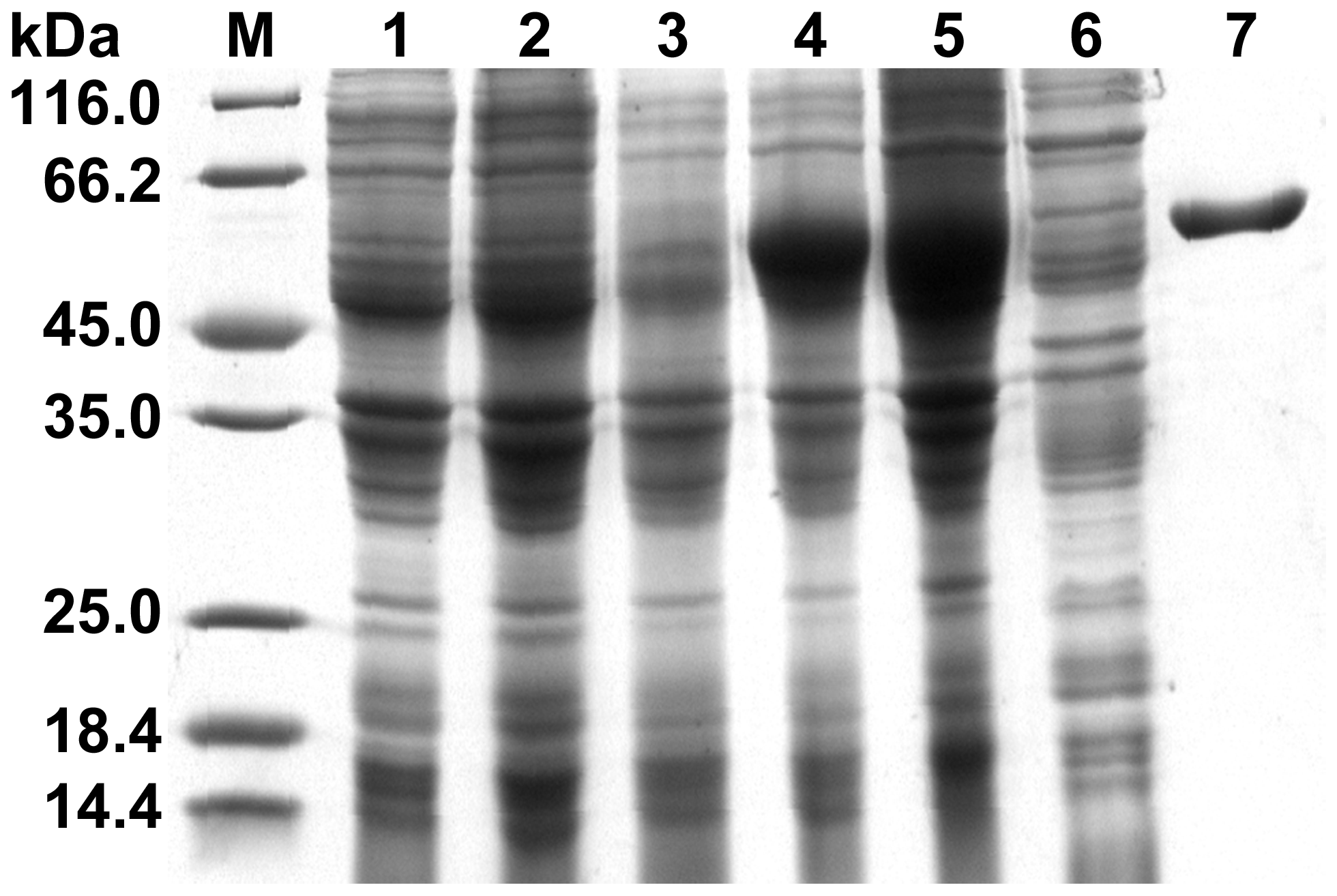


**Figure S1. Expression and purification of r*Cs*HK by 12% SDS-PAGE.** r*Cs*HK was expressed in *E. coli* with IPTG induction and was purified by His-band resin chromatography. Protein markers (lane M), lysate of *E. coli* with pET-28a (+) without induction (lane 1) and with induction (lane 2), lysate of *E. coli* with pET-28a (+)-*Cs*HK without induction (lane 3) and with induction (lane 4), supernatant (lane 5) and precipitant (lane 6) of lysate of *E. coli* with pET-28a (+)-*Cs*HK with induction, and the purified recombinant *Cs*HK (lane 7).
