# Supplementary material for: Sequence Analysis and Molecular Characterization of Clonorchis sinensis Hexokinase, an Unusual Trimeric 50-kDa Glucose-6-Phosphate-Sensitive Allosteric Enzyme
Source: PLoS One. 2014 Sep 18;9(9):e107940. doi: 10.1371/journal.pone.0107940 (PMC4169440; doi:10.1371/journal.pone.0107940)
Supplement: Figure S2 — Identification of r Cs HK by MS. The peptide mass spectra of the purified recombinant protein were obtained on an ABI 4800 Proteomics Analyzer MALDI-TOF/TOF, interpreted and processed using the GPS Explorer software. The identified peptides were matched to those of the hexokinase of C. sinensis [gi|358253389] in the NCBI database. (DOC) [file pone.0107940.s002.doc]

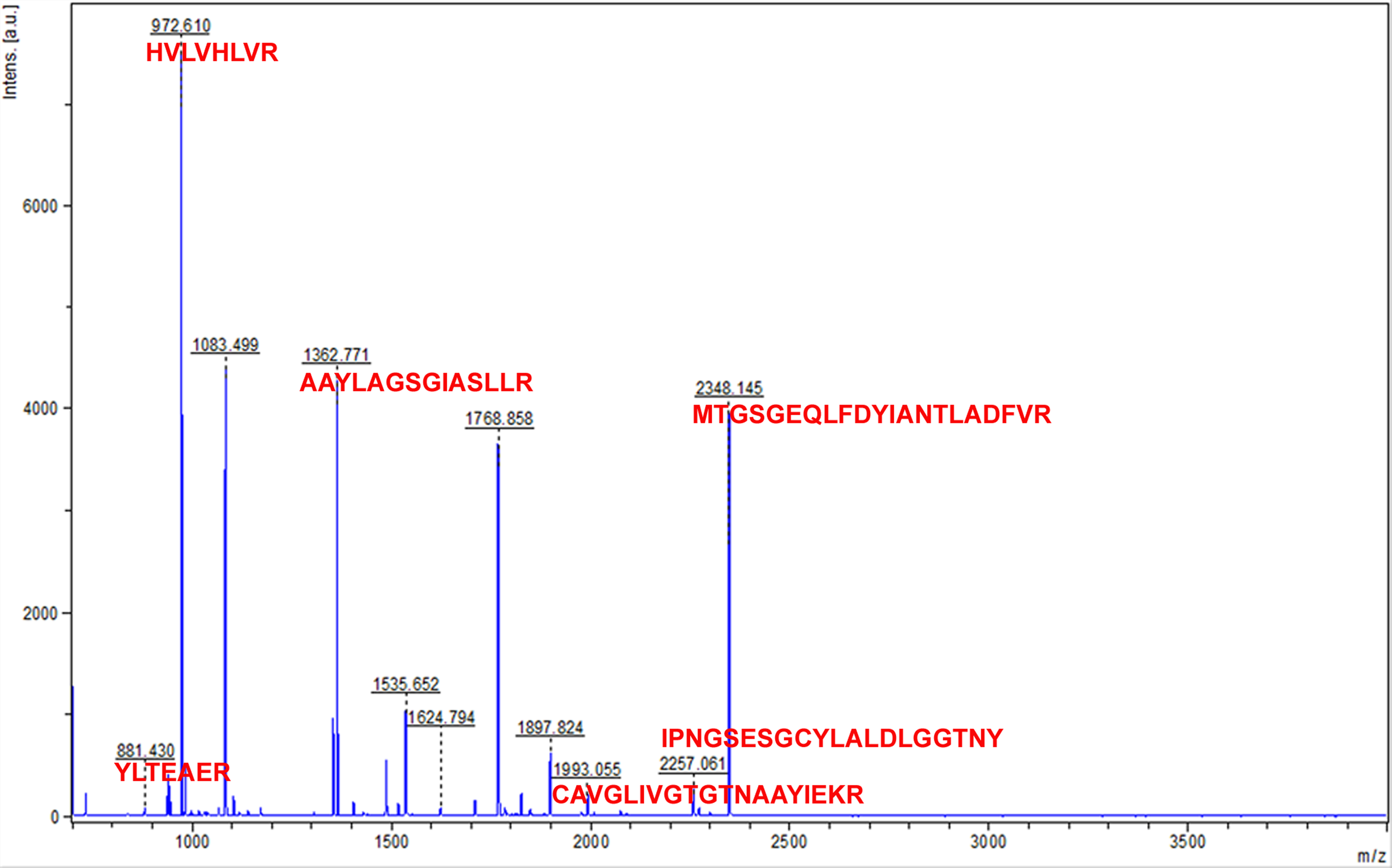


**Figure S2. Identification of r*Cs*HK by MS.** The peptide mass spectra of the purified recombinant protein were obtained on an ABI 4800 Proteomics Analyzer MALDI-TOF/TOF, interpreted and processed using the GPS Explorer software. The identified peptides were matched to those of the hexokinase of *C. sinensis* [gi|358253389] in the NCBI database.
