# Supplementary figures and images for: Sequence Analysis and Molecular Characterization of Clonorchis sinensis Hexokinase, an Unusual Trimeric 50-kDa Glucose-6-Phosphate-Sensitive Allosteric Enzyme
Source: PLoS One. 2014 Sep 18;9(9):e107940. doi: 10.1371/journal.pone.0107940 (PMC4169440; doi:10.1371/journal.pone.0107940)

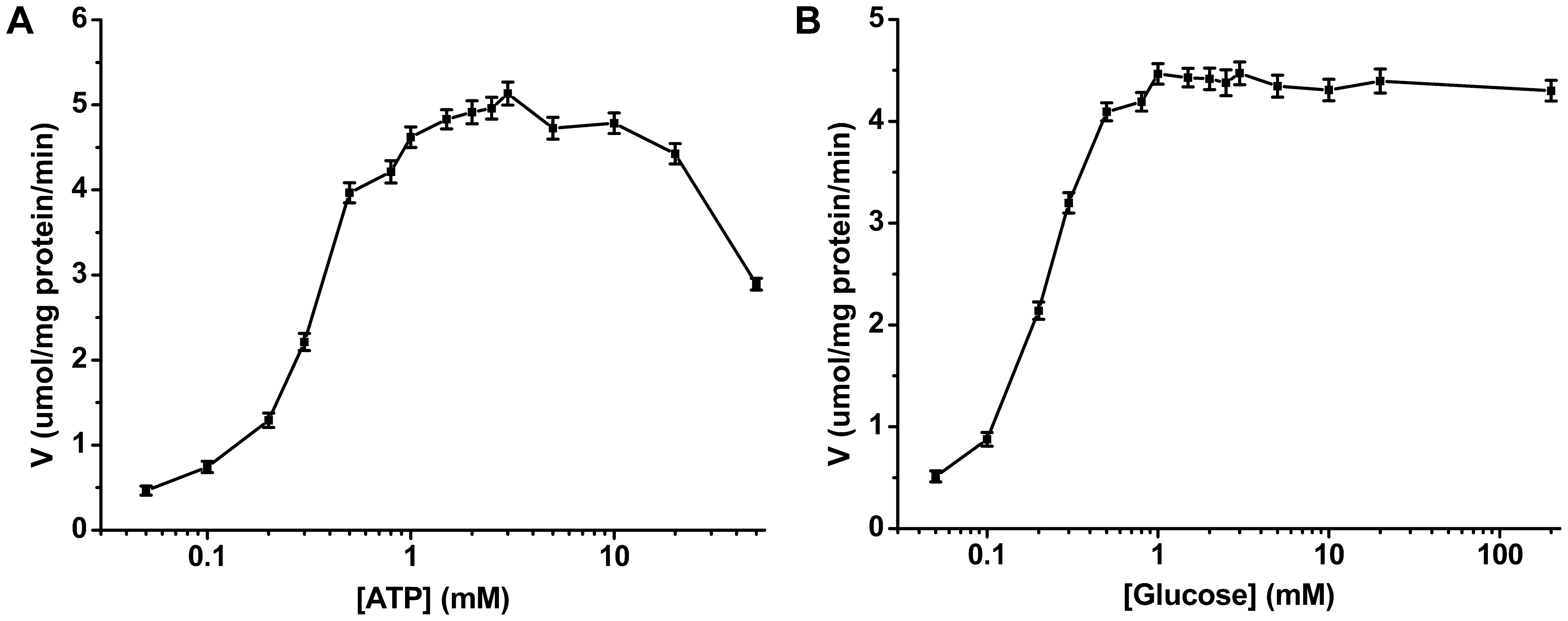


**Figure S3. The substrate saturation curves for r*Cs*HK substrates, including ATP and glucose.**

Supplement: Figure S3 — The substrate saturation curves for r Cs HK substrates, including ATP and glucose. (DOC) [file pone.0107940.s003.doc]
